# Exploring the factors influencing pharmacists' therapeutic reasoning in decision-making: a concurrent think aloud and semi-structured interview study

**DOI:** 10.1007/s11096-026-02216-x

**Published:** 2026-09-18

**Authors:** Daniel Rainkie, Zachariah Nazar, Pim Teunissen, Karen Könings

**Affiliations:** 1 Faculty of Health, College of Pharmacy, Dalhousie University, 5968 College Street, PO Box 1500, Halifax, NS B3H 4R2 Canada; 2 School of Health Professions Education, Maastricht University, Maastricht, The Netherlands; 3 College of Pharmacy, QU Health, Qatar University, Doha, Qatar; 4 Department of Obstetrics & Gynecology, Maastricht University Medical Center, Maastricht, The Netherlands; 5 School of Health Sciences, University of East Anglia, Norwich, UK

**Keywords:** Clinical decision-making, Education, pharmacy, Pharmacists, Prescribing, Template analysis, Therapeutic reasoning, Think-aloud

## Abstract

**Introduction:**

Models of therapeutic reasoning conceptualize how pharmacists apply knowledge to weigh the benefits and risks of treatment options for patients but provide limited detail on the specific factors considered during the process.

**Aim:**

To explore the factors that prescribing pharmacists consider during therapeutic reasoning when making treatment decisions and how those factors are structured hierarchically.

**Method:**

This qualitative study explored the therapeutic reasoning of eight prescribing pharmacists as they worked through three primary care cases selected from hypertension, diabetes, heart failure and pediatric fever. Participants completed a think-aloud protocol to verbalize their thoughts in real time, a post-encounter form to promote reflection, and a semi-structured interview to clarify and elicit additional reasoning considerations. Data were audio-recorded, transcribed, and analyzed using template analysis. Each template iteration was critically reviewed by the research team with a detailed audit trail. Information power was reached at 18 cases (six pharmacists), with the remainder used for confirmation.

**Results:**

We identified 35 factors across five themes: treatment planning, evidence and experience, drug-related factors, patient-centered factors, and setting influences. Therapeutic reasoning typically started with treatment planning to list broad options available in context and rule out options based on contraindications, cost and coverage. The remaining tailored list of options were evaluated using a non-linear process informed by evidence and experience, drug-related factors, and patient-centered factors. Setting influences, such as external pressures, access to clinical decision support systems, or the preferences of other clinicians influenced how the pharmacist considered treatment options. In total, 11 factors were associated with ruling out options, while the remaining factors informed the relative ranking of viable alternatives. Two levels of reasoning were observed: generic reasoning (case-independent) which relied on knowledge alone and patient-specific reasoning where relevant knowledge was applied to the patient case. Dynamic prioritization was observed as factor importance varied by case.

**Conclusion:**

This study advances the conceptualization of therapeutic reasoning by specifying the factors and their functional roles within decision-making. These findings provide a practical foundation for teaching, assessing, and supporting structured clinical decision-making in pharmacy practice.

**Supplementary Information:**

The online version contains supplementary material available at https://doi.org/10.1007/s11096-026-02216-x.

## Impact statements

- Therapeutic reasoning involved weighing treatment options using 35 factors in 5 themes. Practicing pharmacists may use these reasoning factors to reflect on therapeutic decisions, identify overlooked considerations, identify knowledge gaps, and guide continuing professional development.
- Educators and assessors can use the identified factors to make explicit both what pharmacists consider during therapeutic reasoning and how they generate, rule out, compare, and select treatment options.
- Researchers can use these findings to refine and evaluate therapeutic reasoning models and how cases can be designed or evaluated across practice settings and levels of pharmacist expertise.

## Introduction

Therapeutic reasoning is the process by which clinicians weigh the risks and benefits of available treatment options to inform treatment decisions, yet our understanding of what factors contributes to the processes of therapeutic reasoning is unclear [1, 2]. A model specific to pharmacist therapeutic reasoning, Pharm-TRv1, describes how this reasoning unfolds at a conceptual level by describing broad domains of patient-, disease- and drug- related knowledge and the connections between them [3]. Three core reasoning processes, patient-disease, disease-drug, and drug-patient reasoning, connect these domains of knowledge and outline the components of reasoning that contribute to making a treatment choice [3]. Pharmacy-specific studies have identified thematic contributions to these core reasoning processes, such as sociocultural, contextual, or medication-related activities, but do not describe what individual factors contribute to these themes [4, 5]. The descriptions of these factors are currently limited due to the narrow context as well as methodological limitations of included studies [3]. Improving our understanding of what factors pharmacist prescribers consider during therapeutic reasoning as they assess the appropriateness of therapeutic options for an individual case has implications for how to teach and scaffold decision-making in pharmacy education, supporting novice prescribers, and informing research into the drug therapy decisions of other health professions. 

The description of the factors that represent therapeutic reasoning have primarily been described in narrow therapeutic contexts such as antimicrobial management reasoning [6]. One study of pharmacists making antimicrobial treatment choices identified 23 specific factors using semi-structured interviews [6]. While some of these factors are generalizable beyond antibiotic prescribing, such as age, financial factors, and severity or trajectory of illness, eight factors are specific to the antimicrobial treatment choice in hospitals, such as past infections or prior exposure to antimicrobials, which limits their relevance to broader prescribing practices. From a general perspective, the pharmacy specific literature remains unreported. Goldszmidt and colleagues proposed 11 general management reasoning considerations that a physician could apply to a case, including eight directly relevant to therapeutic reasoning such as considering the consequences of treatments on comorbid illnesses, the implications of available treatments and resources, and broadly weighing alternative treatment options taking into account patient preferences [7]. Empirical studies that have used the Goldszmidt framework to assess reasoning identified limited verbalization of these factors ranging from one or two of the eight therapeutic reasoning considerations using think aloud procedures [8, 9]. These studies offer a useful partial understanding of the factors that contribute to therapeutic reasoning, however, also have methodological limitations.

Think aloud approaches are the most common method of data collection used to explore the reasoning of health professionals [10]. Studies which use concurrent think aloud methods represent a participant’s working memory but do not fully reflect background knowledge [11, 12]. Considering that decision making is also hypothesized to be primarily based on pattern recognition and that think aloud studies are the verbal representations of a participant’s working memory, different methodological approaches could identify additional reasoning factors [13]. The addition of follow-up retrospective think aloud, where participants describe their thoughts after a task; post-encounter forms where participants write free text responses to guiding questions or a semi-structured interview with probing questions have been proposed to capture reasoning not identified by think aloud procedures [10, 14, 15]. Studies of pharmacists making treatment decisions which have used both a concurrent think aloud and either retrospective think aloud or semi-structured interview methods have previously addressed the process of reasoning, rather than reasoning factors [16, 17]. As a result, the question of what factors pharmacists consider during therapeutic reasoning remains underexplored.

## Aim

To explore the factors that prescribing pharmacists consider during therapeutic reasoning when making treatment decisions and how those factors are structured hierarchically.

## Method

This qualitative study explored how pharmacist prescribers reasoned through three primary care cases using concurrent think aloud procedures, post-encounter forms documenting decisions, and follow up semi-structured interviews. This complementary approach to data collection allowed us to explore the working memory of pharmacists, promote reflection-in-action, as well as to clarify and elicit reasoning considerations that may have been implied but not verbalized, such as background knowledge. Think-aloud procedures were designed based on methodological guides [10, 12]. Data were analyzed using template analysis within a constructivist perspective. This study is reported in accordance with COREQ guidance (Appendix A) [18].

### Researchers’ positionality

The experiences, perspectives, and professional roles of the interprofessional team influenced what we noticed, valued, and how we interpreted the data in this study. Our perspectives are that therapeutic reasoning includes the interpretation and application of knowledge that is developed by a shared understanding within the individual pharmacist, the larger pharmacy profession, and within the context of a healthcare team [3, 19]. Studying therapeutic reasoning in this way recognizes that decisions are shaped by the pharmacist’s knowledge and how they interpret and prioritize information as they apply that knowledge in their context.

Our research team consists of both insider and outsider perspectives. DR is a pharmacist and is pursuing a PhD in health professions education with extensive experience teaching and mentoring pre- and post-licensure pharmacists. ZN is a pharmacist with a PhD who also has experience teaching and mentoring pharmacy students and pharmacists. PT is a practicing gynecologist with a PhD, whose research activities explore different professionals’ reasoning processes and the way in which they communicate them to reach treatment decisions. KK is a cognitive psychologist with a PhD, whose interest is to understand perspectives of different stakeholders in education to improve the quality of education.

### Participants and recruitment

Pharmacists were eligible to participate if they were licensed in Canada; worked in a community pharmacy, primary care clinic, or specialty ambulatory care clinic; had independent prescribing authority; had prescribed a medication at least once in the past month; and self-assessed as competent to prescribe for patients with hypertension, type 2 diabetes, or heart failure. We anticipated that 5–10 participants solving three cases would provide adequate information power given the focused study aim, theoretically informed design, participants’ rich experience, depth of dialogue, and planned analysis allowing for the identification of the factors present during therapeutic reasoning [20]. Previous studies exploring therapeutic reasoning have included 10–20 participants however, they typically examined a single case or explored reasoning more generally without a case stimulus [6, 8, 21].

Pharmacists were invited by email through the researcher’s professional networks with snowball sampling. Pharmacists self-screened according to the eligibility criteria and informed consent was obtained using online surveys. Participants were offered a $100 gift card as an incentive for their participation.

### Case development

Four common primary care cases, each with several reasonable treatment options, were developed to allow pharmacists to deliberate between treatment options. Each case was designed to be unambiguous, well-defined, and include the necessary subjective, objective, and laboratory data to make a treatment choice, allowing participants to focus on therapeutic reasoning rather than diagnostic reasoning, or problem identification. Adult type 2 diabetes, hypertension, heart failure, and pediatric fever cases were developed by DR, who has extensive experience designing cases for classrooms and simulation for undergraduate and licensed pharmacists. Initial drafts were reviewed by two pharmacists and a physician for fidelity, appropriate complexity for the setting, and completeness of information required to make a therapeutic decision. Revisions included wording changes for clarity and the addition of further laboratory results. Summary of cases are found in Box 1.

**Box 1:** Summarized description of the hypertension, diabetes, heart failure and pediatric fever cases

Hypertension: a 56 year-old Scottish male who presents to your primary care clinic with mild bilateral pedal edema and wishes to have better blood pressure control. He has a history of uncontrolled hypertension on amlodipine 5 mg once daily, mild asthma controlled with budesonide and formoterol, normal renal function, normal electrolytes, with all other labs are stable.
Diabetes: a 71 year-old Japanese female who presents with uncontrolled diabetes with an A1C of 9.1% without symptoms. She has a history of a myocardial infarction, chronic kidney disease with mild albuminuria and an eGFR of 49, knee osteoarthritis controlled with acetaminophen, and controlled hypothyroidism. All of the investigations are normal, including electrolytes and stable serum creatinine.
Heart failure: a 65 year-old Jordanian male who presents to your primary care clinic for a regularly scheduled follow up. He has a history of coronary artery disease, hypertension, and heart failure with reduced ejection fraction with good symptom control, and all labs are within normal range with room to move. There are opportunities to adjust several of his heart failure therapies to achieve his goals.
Pediatric fever: a 20-month-old child who presents to your pharmacy for analgesic and fever management secondary to a presumptive viral otitis media that has been assessed by a physician. There are no red flags, the child is eating a drinking a bit less than usual, has been having a normal number of wet diapers, and has not tried anything in the past.

### Data collection

Eligible pharmacists were invited to complete the think aloud and semi-structured interviews between March and July 2024. In preparation for the think aloud, DR introduced the idea of thinking aloud and participants completed two non-clinical related warm up exercises by asking them to verbalize their thoughts while identifying the eighth letter after ‘E’ in the English alphabet and calculating the answer to 12 × 17. DR provided feedback to familiarize participants with the expected think-aloud process. DR then shared one therapeutic reasoning think aloud example unrelated to the cases as an example. Pharmacists were provided all three cases in an email with the first two cases being a selection of hypertension, type 2 diabetes, or heart failure, followed by pediatric fever as the third case to identify if reasoning differed for cases with remote expertise. Pharmacists were instructed to pay special attention to when they thought about different treatment options as they sequentially solved each case. If necessary, pharmacists were prompted after 15 seconds of silence to “please think aloud.” If at any point a pharmacist self-assessed that a case was outside their competence to prescribe (e.g. heart failure), an alternative case was provided.

Immediately after each case, pharmacists completed a 6-item online post-encounter form which included items to promote reflection-in-action: their summary of the primary case concern; what therapeutic options that were immediately ruled out with a brief rationale; if any additional information that the pharmacist would seek and what that would be; their final treatment decision; if they would be comfortable writing this prescription (yes/no); listing one or two alternative therapeutic options that were considered as their next best choice with a brief rationale.

An individualized semi-structured interview was completed within seven days of the think aloud procedures using data from their think-aloud transcripts and post-encounter forms. For example, treatment options that were identified in the think-aloud or documented in the post-encounter form were used as prompts during the interview. This allowed the interviewer to explore how the participant’s selected prescription compared with options that were considered but ultimately not chosen. The structure of the interview began with an overview of DR’s interpretation of the participant’s reasoning process, followed by a detailed discussion of how their chosen prescription compared with alternative options including class vs class considerations (e.g. SGLT2 inhibitors vs GLP-1 agonists) and within class drug vs drug considerations (e.g. empagliflozin vs dapagliflozin).

Interviews were conducted virtually on Microsoft Teams, transcribed verbatim and then pseudonymized for confidentiality. The semi-structured interview guide was developed, desk validated, and piloted before implementation. The pilot followed the complete study procedure and led to the removal of questions and probes that elicited repetitive responses, as well as revisions to the wording of several items. The base semi-structured interview guide is provided in Appendix B.

### Data analysis

Template analysis was used to code the think aloud and semi-structured interview transcripts using the software Dedoose v10 (Los Angeles, CA: SocioCultural Research Consultants, LLC) [22]. Compared with inductive thematic analysis, template analysis emphasizes a more detailed and hierarchical coding. The initial a priori template was based on the management reasoning completed by Goldszmidt et al. and contained 11 items [7]. We renamed ‘management reasoning’ to ‘therapeutic reasoning’ to focus on drug and non-drug therapeutic options. We inductively identified and coded factors from think aloud and semi-structured interview transcripts and post-encounter forms. The template analysis codebook was iteratively developed with a detailed audit trail by DR with a detailed, critical review by the research team in between versions as suggested by King [22]. The final (fifth) version of the codebook was finalized according to consensus within the research team and then applied to the full data set by DR. Adequate information power was judged to be reached at 18 cases (six pharmacists), with the remainder used for confirmation.

### Ethics approval

This study was performed in line with the principles of the Declaration of Helsinki. Approval was granted by Dalhousie University (REB# 2024–7024).

## Results

Eight practicing pharmacists were recruited from two Canadian provinces with similar scopes of practice, Nova Scotia (n=6) and Alberta (n=2). Five pharmacists had at least 10 years of experience and three had fewer than 10 years. Four pharmacists had been independently prescribing for at least five years and four had been prescribing for less than five years. In the final dataset, seven hypertension, eight diabetes, one heart failure, and eight pediatric fever cases were included. Participants took a median of 46 min to think aloud through all three cases and complete the post-encounter forms (range 30 to 72 min) and the median semi-structured interview duration was 58 min (range 38 to 66 min).

We identified 35 discrete considerations, or reasoning factors, that participants considered when evaluating treatment options throughout the cases. Of the original template of Goldszmidt’s 11 management reasoning factors, six were still present in the final template. The factor “weigh alternative treatment options taking into account patient preferences” was vastly expanded. Five overarching themes, each containing 3 to 12 factors, were identified: (1) treatment planning; (2) evidence and experience; (3) drug-related; (4) patient centered, and (5) setting influences. We identified a general pattern that pharmacists started their reasoning by outlining the intended treatment goals and listing a filtered set of prescription options before engaging with reasoning factors from themes 2, 3, and 4. Identified themes, factor descriptions, and illustrative quotes are presented in Table 1, with a detailed description provided in Appendix C. For transparency, the presence of each factor, and whether it was identified from the think-aloud or semi-structured interview data, are documented in Appendix D.

**Table 1:** Themes, factors, and illustrative quotes during pharmacist therapeutic reasoning

Theme	Factor	Description
Treatment Planning	Establish goals of care	Treating symptoms, improving function, altering prognosis or cure; taking into account patient preferences, perspectives, and understanding. Includes both patient specific goals of therapy and clinician goals of therapy.
	Listing options/indications	Listing relevant treatment options for a disease state (e.g. medication indicated to treat disease or a compelling indication).
	Non-pharmacological treatment approaches	Considering non-pharmacological co-interventions as part of management plan.
	Contraindications	Initial ruling out of treatment option(s) based on contraindications such as: Allergies, drug/class specific contraindications.
	Cost and coverage	Treatment is on provincial (public payer) formulary. Co-pay treatment is acceptable.
Evidence and Experience	Evidence of efficacy	The clinician's understanding of the evidence (or lack of evidence) of therapeutic effect for defined outcomes.
	Evidence of safety	The clinician's understanding of evidence (or lack of evidence) of treatment harms.
	Experience as evidence	Clinician draws on their own experiences using a medication as a source of efficacy or safety evidence.
	Evidence strength or robustness	The clinician’s judgement or critical appraisal of the strength/weakness of evidence and robustness/limitations of evidence regarding an efficacy or safety outcome.
	Evidence generalizability and applicability	The clinician's judgement of the ability or inability of being able to apply evidence to the patient case or extrapolating evidence beyond populations.
Drug Related Factors	Route of therapy	General acceptability of medication route of administration influences choice (e.g. oral, SC, IM, rectal).
	Dose adjustments for organ function	Adjusting dose or frequency based on organ function (e.g. renal or liver function).
	Regimen complexity and flexibility	Factors affecting complexity and flexibility of a regimen such as titrating to target doses, tapering considerations, flexibility to use combination products, or risks of administration errors due to interval between doses.
	Duration of action	Clinician considers a preference for drugs with a longer duration of action.
	Onset of action	Clinician considers the onset of action in relation to the intended treatment effect. Drugs with a faster onset of action may not be relevant for chronic conditions.
	Drug-interactions	Perceived risk of drug interactions, in general or actual presence of drug interactions based on patient's current medications.
	Availability and shortages	Availability of a treatment option and history of availability (drug shortages).
	Mechanism of action and pharmacodynamics	Medications preferred that more specifically targets pathophysiology of problem (medical problem or side effect problem).
	Dose efficacy and equivalency	Evaluating whether different doses of the same or different medications within or across drug classes achieve equivalent therapeutic effects, particularly when switching between agents.
	Formulations	Assumed or acknowledged acceptability of medication formulation influences choice (tablet, liquid, suspension, extended release, etc.).
	Environmental impact	Environmental impact of treatment option (e.g. packaging, MDI vs DPI).
Patient Centered Factors	Disease severity	More severe disease presentation necessitates specific treatment interventions (e.g. insulin which carries higher risk) or combination treatment.
	Disease trajectory and long term chronic and complication considerations	Considering possible complications of uncontrolled disease and risk factors for comorbidities over time based on initial diagnosis (e.g. hypertension diagnosis and risk of chronic kidney disease; diabetes and risk of cardiovascular disease).
	Risks of polypharmacy and prescribing cascades	Recognizing the presence of or avoiding polypharmacy or prescribing cascades.
	Patient specific tolerability/side effect preferences	Patient specific comments regarding assessment of overall tolerability, including drug cautions, that would require monitoring for that specific disease state being treated (e.g. HF and dapagliflozin and fluid status).
	Actual response to current or previous treatments	Adjustment of options based on therapeutic or safety response to current or previous treatments (including patient noticeable changes such as weight loss, QoL improvement, etc.).
	Impact of management on comorbid illnesses	Treatment recommendation improves or worsens comorbid illness or complicates patient assessment.
	Monitoring burden (patient)	Laboratory investigations required to monitor for safety of medication.
	Patient preference	Involving patient in shared decision making when additional input or preferences are needed.
	Psychosocial context	Describing how social history or assessment of patient’s health literacy influences decision making, or availability of resources/support at home (e.g. caregivers).
	Age considerations	Age as a risk factor for safety concerns, for other diagnoses, for long term trajectory and common comorbidities.
Setting Influences	Available healthcare resources	Clinician considers system specific use of resources regarding the treatment or monitoring of patients (i.e. cost of monitoring, availability of monitoring, HCP access).
	Support from clinical decision support systems	Input from external support systems to provide specific drug information as a passive source of information (e.g. flagging drug interactions, selecting drug dose or strength, identifying dosage forms).
	External influences on prescribing practice	Acknowledging local influences on practice such as local prescribing patterns, physician/NP preferences, acknowledged marketing, and available work time.

### Treatment planning

Early in the reasoning process, participants oriented themselves with the goals of care and treatment priorities to move from a generic list of treatment options for a medical problem to a narrower list of potential options. For example, several pharmacists noted both clinician goals and patient goals, stating:

“we need to get her blood sugar down, otherwise her kidney function is going to continue to be compromised [clinician goal] and that would increase her risk of subsequent cardiac events [patient provided goal]” (RPh06).

The elimination of options was noted to be based on contraindications and cost or coverage concerns. For example, two pharmacists noted,

“ …beta blocker not an option because of the asthma,” (RPh05) and “I mean couple options if coverage is not an issue. If she has insurance and could get covered for it” (RPh08).

Ultimately, these treatment planning factors were most prevalent near the start of reasoning and were less prevalent towards the end of decision making. When used later in reasoning, these factors were more associated with double checking the feasibility of options. The remaining option(s) were taken forward and evaluated according to the factors found in the evidence and experience, patient-centered, and drug-related themes.

### Evidence and experience

Participants used factors related to evidence and experience to differentiate options based on their effectiveness towards the set goal(s) and their risk of harms. Pharmacists judged the efficacy and safety of treatment options by drawing on their understanding and interpretation of the available evidence and their prior clinical experience. For example, one pharmacist noted:

“I’m really looking for a med that has additional cardiac benefits. So SGLT2 or a GLP1 agonist, with my preference being a GLP1 agonist because we’re more likely to get her to target A1c” (RPh04).

As participants compared different medication classes or individual agents, they weighed the strength and robustness of the evidence, or the lack thereof:

“From what I understand is we have some evidence for some of the GLP1s, but not all of them yet. Like we are assuming it’s gonna be a class effect that I still think we’re waiting on a little bit more information” (RPh05).

Participants drew on evidence of efficacy and safety in two different ways. One was a general interpretation of the evidence and the other was making patient-specific judgments about fit with the individual’s goals and preferences. When pharmacists employed these factors during reasoning they often followed up by considering if the drug remained viable. For example, as a pharmacist considered empagliflozin and liraglutide, they considered the evidence for efficacy to be stronger for empagliflozin and was the preferred option for that case.

### Drug-related factors

Within this theme, participants considered the defining features of a medication or product such as available routes of therapy, dosing or regimen complexity, as well as drug-specific characteristics such as it’s pharmacokinetic, pharmacodynamic, and drug-interaction profile to evaluate potential treatment options. Pharmacists generally described facts regarding individual medications such as titration or tapering complexities, duration or onset of action, mechanism, environmental impact, or medication availability or shortages:

“Otitis media is inflammation and I'm choosing an anti-inflammatory” (RPh01).

“...you know, in rural [pharmacies] you might be looking at availability a little bit more.” (RPh05)

These facts impacted relative ranking between treatment options but was not identified to rule out any treatment options. Several factors were centered around product specific characteristics and their general gathered, assumed, or inferred patient acceptability. These included various treatment characteristics such as route of therapy (e.g. oral or injection), formulation (e.g. tablet or liquid), future or immediate dose adjustments for organ function (e.g. renal function), regimen complexity, and drug interactions with a patient’s current drug regimen:

“I think like just cause like with the SGLT2 it's like a one and done dosing you don’t really have to check in with them again” (RPh08),

### Patient-centered factors

Patient-centered factors described how participants assessed options based on patient-specific information and their judgement or assessment of the patient’s experiences, history, or preferences. This included the patient’s actual or previous response to treatment, side effect preferences, impact of treatment option on comorbid illnesses, monitoring burden, age considerations, the psychosocial context, and risk of prescribing cascades. One pharmacist weighed the competing risks of hypoglycemia between options by understanding the patient’s context while another commented on monitoring:

“...depending on it doesn't say like what her home situation is, or what's going on like does she have a partner at home?” (RPh05)

“So his blood pressure has got lots of room to wiggle these meds up. His creatinine clearance has lots of room to move” (RPh02)

These factors helped participants judge whether a treatment that appeared clinically appropriate was also appropriate for the individual patient in practice.

### Setting influences

Pharmacists described how external influences on their practice, availability of system level resources, and clinical decision support systems influenced how options were being considered. Local prescribing patterns, industry marketing, and time to make a decision were acknowledged as external influences of prescribing with comments such as marketing, local prescriber preferences, and time in clinic. The availability of resources was considered by pharmacists when thinking about rural or urban centers and the cost of these resources such as access and cost to laboratory tests for monitoring drug therapy. Clinical decision support systems facilitated the pharmacist’s decision making with access to information or other supports.

### Dynamic importance of factors

During analysis, we identified that participants used similar factors differently between cases, as if each factor existed on a continuum of importance. When a factor reached some perceived threshold of importance to the pharmacist, it was used to rule out an option. Four of the five themes had at least one factor that was associated with ruling out options. These factors were: cost and coverage, contraindications, evidence of efficacy, evidence of safety, route of therapy, impact on comorbidities, illness severity, response to current or previous therapies, shared decision-making, and patient preferences. For example, cost, coverage, route of therapy, and illness severity was used to exclude options. Likewise, if a factor did not reach the threshold of importance, that factor influenced the relative ranking of otherwise viable alternatives.

### Connections between themes

Participants exhibited their reasoning in a non-linear manner and fluidly moved back-and-forth between evidence and experience, patient, and drug factors throughout their reasoning. Participants verbalized a chain of reasoning when concluding if a treatment option was viable and it’s relative ranking between options. For example, during the hypertension think aloud, a pharmacist identified the goals of therapy followed by considering the patient factors of disease severity and trajectory which then led to list options. After they considered the efficacy of two options and considered the likely tolerability of those medications according to patient lab values which led to considering drug interactions before revisiting the list of options and considering other factors relevant to a different medication. Reasoning was identified to start from any of the themes, but the treatment planning theme was the most common. 

Throughout their reasoning, pharmacists included elements of metacognition such as identifying their own knowledge gaps, scrutinizing the quality of the case information available, addressing uncertainty, summarizing their rationale, and controlling the planning of next steps. Several pharmacists highlighted alternate scenarios where different options would be considered if compelling indications were present. Before being satisfied with their reasoning and exiting their therapeutic reasoning process, many pharmacists performed a double check before revisiting their list of options:

“I’m thinking OK, did I miss anything? I will re-review my process” (RPh01)

## Discussion

We used concurrent think-aloud procedures, post-encounter forms, and follow up semi-structured interviews to explore pharmacists’ therapeutic reasoning as they worked through common primary care problems. We identified 35 reasoning factors that were organized into five themes: treatment planning, patient factors, drug factors, evidence and experience factors, and setting influences. Importantly, not all factors contributed equally to decision making and different factors were used according to the clinical context. These results provide an important step toward understanding the content and complexity of therapeutic reasoning in pharmacy practice.

### Comparing factors of therapeutic reasoning

From a context-specific perspective, our findings suggest that therapeutic reasoning in broader primary care pharmacy practice involves a wider range of considerations including evidence and experience and drug factors than would be identified in a narrower context. Several factors identified in this study overlap with those reported by Gruenberg and colleagues when making antibiotic treatment decisions [6]. Shared considerations included patient characteristics like age and allergies and treatment-related factors such as prior exposure to antimicrobials and the number and frequency of medication doses required throughout each day. Gruenberg identified more contextual factors that affected decisions such as local antibiograms and institution specific practices or policies [6]. These observations suggest that disease-specific and broader primary care studies make complementary contributions to understanding therapeutic reasoning by identifying both how reasoning is shaped by particular clinical contexts and which considerations recur across areas of practice. Recent international research also demonstrates that pharmacist-clinician roles are variably defined and enacted and depend on interprofessional and organizational conditions [23].

From a general perspective, a 2025 study of senior postgraduate medical trainees identified eight similar factors such as patient goals, age, comorbidities, prior treatment response, cost and coverage, and social considerations [24]. We identified similar factors, but with different emphasis. For example, our study further describes the evidence and guidelines factor identified in the physician study as evidence for efficacy, safety, strength of evidence, and its generalizability or applicability to the patient context [24]. As expected, drug characteristics considered during reasoning were importantly different between physicians and pharmacists. In the study of physicians, ‘drug characteristics’ included a description of route of administration and side effects [24]. In our study we identified several additional drug-centered factors including drug interactions, dosing, onset and duration of action, regimen complexity, and availability. In Parsons’ study of physicians, provider factors such as risk tolerance, team dynamics, and cognitive biases were prominent, but not identified in our study. In aggregate, these findings suggest that some therapeutic reasoning factors are shared across professions, while others are either profession-specific or dependent on the stimuli used to explore reasoning.

### Assessment, non-linearity, and metacognitive double check

From an assessment perspective, learners are often assessed on the flow, organization, or systematicity of their thought process [25]. Related literature specifically note “disturbed thought process” or “lapses and slips” as errors of reasoning [5]. While pharmacists in our study commonly ruled out options early in the therapeutic reasoning process, our findings suggest that the subsequent weighing of treatment options and engaging with therapeutic reasoning factors is non-linear. This is consistent with studies in both the physician and other pharmacy literature [4, 9, 26]. Collectively, these findings call into question the emphasis on linearity and systematicity when assessing therapeutic reasoning. Although a simplified, linear, sequential processes might be helpful in teaching therapeutic reasoning, it does not seem reflective of the processes used by professionals.

In our study, we identified that pharmacists engaged with metacognitive double-checking both in response to uncertainty and near the end of the reasoning process, to determine whether they were satisfied with their evaluation of treatment options and whether anything had been missed or overlooked. Our findings are similar to Walker and colleagues who also identified that pharmacy students commonly double checked their thinking when they experienced uncertainty [26]. This suggests that educators should explicitly teach, demonstrate, and emphasize metacognitive double checking before learners finalize a treatment decision.

### Impact on Therapeutic Reasoning Models and Future Research

The results of this study complement the recently proposed Pharm-TRv1 model by identifying treatment planning as a key overarching process as well as elaborating on several therapeutic reasoning subprocesses within the core patient-disease, disease-drug, and drug-patient processes [3]. For example, significant additions to the drug-patient reasoning processes include previous treatment response, monitoring burden, physiological limits, applicability of evidence, and many drug centric considerations such as adjustments for organ function, drug interactions, formulation, regimen complexity, and risk of prescribing cascades. Additionally, we identified a treatment planning phase which was not identified in the original model. For learners and educators, our additions may help make therapeutic reasoning more explicit by identifying factors of reasoning that can be taught, observed, and discussed during feedback. Additional research is also needed to generate validity evidence for the application of the updated Pharm-TRv1 across different practice settings, such as hospital pharmacy. For practicing pharmacists, the model may also provide a structure for reflection, identifying knowledge gaps, and informing continuing professional development focused on therapeutic decision-making.

### Limitations

Although the methods were judged to provide adequate information power, it is unlikely that all possible therapeutic reasoning factors could be identified within this study. Firstly, the participants self-selected into the study and were recruited through purposive and snowball sampling. This approach may have favoured pharmacists with greater confidence, experience, or interest in therapeutic decision-making, as well as participants from similar professional networks. Our findings may therefore be most transferable to contexts in which pharmacists have similar responsibilities, experience, and scopes of practice.

The cases used were representative of common problems encountered in primary care; therefore, exploring a broader range of clinical scenarios may identify additional reasoning factors. Our study included well-defined cases to minimize ambiguity and allow the participants to focus on making treatment decisions. Therefore, our findings do not represent how pharmacist prescribers integrate their broader responsibilities of clinical assessment and problem identification, or their diagnostic reasoning, with their therapeutic reasoning [27]. Real world or ill-defined cases incorporating missing or conflicting information may further characterize this relationship. The case stimuli for this study were paper based and do not reflect the communication and clinical skills needed to gather necessary information and make judgements about the quality and adequacy of information. Patient preferences were clearly provided in the cases and how pharmacists elicit and incorporate these preferences may impact reasoning. Therefore, therapeutic reasoning may differ when explored in the broader perspective of clinical reasoning [28].

We did not systematically compare prescribing decisions between participants or assess their concordance with current evidence and clinical guidelines. Future research could build on these findings to compare reasoning factors and evaluate the appropriateness of decisions between pharmacists. Finally, our findings are representative pharmacist prescribers making independent decisions. It is possible that these factors may be shared or delegated within team-based decision-making contexts, such as hospital rounds or specialty ambulatory clinics involving multiple healthcare professionals.

## Conclusion

This study further characterises the factors that pharmacists consider during therapeutic reasoning. It identifies 35 factors across five themes: treatment planning, evidence and experience, drug factors, patient-centered factors, and setting influences. Reasoning typically begins with treatment planning to list general options and rule out options based on patient-specific contraindications and cost and coverage. Pharmacists then compare the remaining options through a non-linear process shaped by factors from the evidence and experience, drug-related considerations, and patient-centered factors.

These findings advance current conceptualizations of therapeutic reasoning by providing a more nuanced understanding of the processes underpinning pharmacists’ treatment decision-making. They also have important implications for pharmacy education and assessment, particularly by challenging overly linear representations of reasoning and highlighting the need to explicitly teach, model, and evaluate the complex and adaptive nature of therapeutic reasoning in clinical practice.

## Supplementary Information

Below is the link to the electronic supplementary material.

Supplementary file1 (DOCX 41 KB)

## Data Availability

The datasets generated during and/or analysed during the current study are available from the corresponding author on reasonable request.
