# Supplementary material for: Exploring the factors influencing pharmacists' therapeutic reasoning in decision-making: a concurrent think aloud and semi-structured interview study
Source: Int J Clin Pharm. 2026 Sep 18;48(6):2383–93. doi: 10.1007/s11096-026-02216-x (PMC13731465; doi:10.1007/s11096-026-02216-x)
Supplement: Supplementary file 1 [file 11096_2026_2216_MOESM1_ESM.docx]

# Supplementary Materials

## Appendix A – COREQ Consolidated criteria for reporting qualitative studies (COREQ): 32-item checklist

Tong A, Sainsbury P, Craig J. Consolidated criteria for reporting qualitative research (COREQ): a 32-item checklist for interviews and focus groups. International Journal for Quality in Health Care. 2007. Volume 19, Number 6: pp. 349 – 357

| **Item No** | | **Guide Questions/Description** | **Reported on Page #** |  |  |
| --- | --- | --- | --- | --- | --- |
| **Domain 1: Research team and reflexivity** | | | |  |  |
| **Personal Characteristics** | | | |  |  |
| 1. Interviewer/ facilitator | | Which author/s conducted the interview or focus group? | 5,6 |  |  |
| 2. Credentials | | What were the researcher’s credentials? E.g., PhD, MD | 4 |  |  |
| 3. Occupation | | What was their occupation at the time of the study? | 4 |  |  |
| 4. Gender | | Was the researcher male or female? | 4 |  |  |
| 5. Experience and training | | What experience or training did the researcher have? | 4 |  |  |
| **Relationship with participants** | | | |  |  |
| 6. Relationship established | | Was a relationship established prior to study commencement? | 5 |  |  |
| 7. Participant knowledge of the interviewer | | What did the participants know about the researcher? e.g. personal goals, reasons for doing the research? | 5 |  |  |
| 8. Interviewer characteristics | | What characteristics were reported about the interviewer/facilitator? e.g. Bias, assumptions, reasons and interests in the research topic | 3,4 |  |  |
|  | **Domain 2: study design** | | |  |  |
|  | **Theoretical framework** | | |  |  |
|  | 9. Methodological orientation and Theory | What methodological orientation was stated to underpin the study? e.g. grounded theory, discourse analysis, ethnography, phenomenology, content analysis | 4 |  |  |
|  | **Participant selection** | | |  |  |
|  | 10. Sampling | How were participants selected? e.g., purposive, convenience, consecutive, snowball | 5 |  |  |
|  | 11. Method of approach | How were participants approached? e.g., face-to-face, telephone, mail, email | 8 |  |  |
|  | 12. Sample size | How many participants were in the study? | 9 |  |  |
|  | 13. Non-participation Setting | How many people refused to participate or dropped out? Reasons? | n/a |  |  |
|  | 14. Setting of data collection | Where was the data collected? e.g., home, clinic, workplace | 9 |  |  |
|  | 15. Presence of nonparticipants | Was anyone else present besides the participants and researchers? | 5-8 |  |  |
|  | 16. Description of sample | What are the important characteristics of the sample? e.g. demographic data, date | 9 |  |  |
|  | **Data collection** | | |  | No |
|  | 17. Interview guide | Were questions, prompts, and guides provided by the authors? Was it pilot tested? | 8, appendix B |  |  |
|  | 18. Repeat interviews | Were repeat interviews carried out? If yes, how many? | n/a |  |  |
|  | 19. Audio/visual recording | Did the research use audio or visual recording to collect the data? | 9 |  |  |
|  | 20. Field notes | Were field notes made during and/or after the interview or focus group? | n/a |  |  |
|  | 21. Duration | What was the duration of the interviews or focus group? | 9 |  |  |
|  | 22. Data saturation | Was data saturation discussed? | 8,9 |  |  |
|  | 23. Transcripts returned | Were transcripts returned to participants for comment and/or correction? | n/a |  |  |
|  | **Domain 3: analysis and findings** | | |  |  |
|  | **Data analysis** | | |  |  |
|  | 24. Number of data coders | How many data coders coded the data? | 9 |  |  |
|  | 25. Description of the coding tree | Did the authors provide a description of the coding tree? | 8,9 |  |  |
|  | 26. Derivation of themes | Were themes identified in advance or derived from the data? | 8,9 |  |  |
|  | 27. Software | What software, if applicable, was used to manage the data? | 8 |  |  |
|  | 28. Participant checking | Did participants provide feedback on the findings? | n/a |  |  |
|  | **Reporting** | | |  |  |
|  | 29. Quotations presented | Were participant quotations presented to illustrate the themes/findings? Was each quotation identified? e.g., participant number | Table 1  Appendix C |  |  |
|  | 30. Data and findings consistent | Was there consistency between the data presented and the findings? | 20-25  Appendix D |  |  |
|  | 31. Clarity of major themes | Were major themes clearly presented in the findings? | 20-25 |  |  |
|  | 32. Clarity of minor themes | Is there a description of diverse cases or a discussion of minor themes? | 20-25 |  |  |

## Appendix B – Base version of the semi-structured interview guide to be personalized according to the participant responses

Thank you for taking part in the think-aloud part of our study and for taking the time from your day to complete this interview. The purpose of this interview is to help clarify some of the cognitive processes that you completed during the think-aloud in order to get the best representation of your thought process possible.

Let’s quickly review case #. [Summary of case; wording as per Box 1].

You had identified (#) options, __list of options__, that you considered to treat this patient and ultimately chose ___(drug of choice)___.

1. I would like to ask further questions about how you selected your recommended therapy.
   - [AAA to summarize think aloud]
   - For you, what makes __list of options__ a top of mind medications?
2. In your post-encounter form, you ruled out __drug__. What are your reasons why this drug was ruled out?
   - How do the benefits of your chosen treatment compare to treatment X?
   - How do the risk of treatment harms of your chosen treatment compare to treatment X?
3. Thinking about other options that you may have automatically not considered, would __list of 2 drugs/combination of drugs not identified in post encounter form or think aloud __ be an option?
4. As you were thinking aloud, you compared __drug vs drug or class vs class__. I’d like to explore how your next best alternative compares to your chosen drug treatment.
   - List factors spoken or written about
     - Ask items that require clarification
   - Ask questions about class vs class or drug comparisons within class for the factors listed above (e.g. Comparatively, do you perceive any important differences between SGLT2s and semaglutide?)
5. Are there any system related factors that influenced your decision, such as cost, availability of a medicine / drug shortages?
6. Are there any support systems that you would rely on receiving information from (e.g. drug-drug interactions from your prescribing software)?
7. Do you think your local practice may have influenced your decision? For example, do you think a pharmacist with similar training and skills to you in the next city over would have made the same decision?

## Appendix C: Themes, Factors, Definitions, and Quotes

| Treatment planning | Establish goals of care | Treating symptoms, improving function, altering prognosis or cure; taking into account patient preferences, perspectives, and understanding. Includes both patient specific goals of therapy and clinician goal of therapy. | 1) "It's hard to say how intensively do you wanna get? Yes, we know the cardiovascular benefit, but it's also you know multi pharmacies, how many medications, do you want to get a complicated routine." (RPh07, hypertension) 2) "we need to get her blood sugar down, otherwise her kidney function is going to continue to be compromised and that would increase her risk of subsequent cardiac events." (RPh06, diabetes) 3) "We can treat that blood pressure to a lower target. I’m thinking 120 to 130 for systolic blood pressure, um and he's pretty close with his current regimen." (RPh01, hypertension) |
| --- | --- | --- | --- |
| Treatment planning | Listing options / indications | Listing relevant treatment options for a disease state (e.g. medication indicated to treat disease or a compelling indication). | 1) "any time I have a patient who's A1c is like 8.5 or above, I think either insulin or GLP1." (RPh08, diabetes) 2) "And then, given her cardiac history, that's going to lead me down the SGLT 2, GLP 1 road versus something like insulin." (RPh04, diabetes) 3) "So for so like the ABCD's of hypertension meds, so like ACE or ARB, so option beta blocker not an option because of the asthma. Calcium channel blocker, we could have switched to another CCB that would have been hmm viable. But you know, they might have still had this swelling again. And then diuretics." (RPh05, hypertension) |
| Treatment planning | Non-pharmacological treatment approaches | Considering non-pharmacological co-interventions as part of management plan | 1) "So you know what can we look at some of her lifestyle and diet maybe to help along with the A1C lowering" (RPh07, diabetes) 2) "Third choice. Umm, not really feasible, but we could really try and maximize like diet and exercise because we know that does more lowering than any medication will." (RPh05, diabetes) 3) "He's only drinking 2 units of beer per week. That's, I would say, not a super significant amount, but if you wanted to do everything that he could to reduce his blood pressure, he could eliminate those and increase his activity" (RPh03, hypertension) |
| Treatment planning | Contraindications | Initial ruling out of treatment option(s) based on contraindications such as: Allergies, drug/class specific contraindications | 1) "With both medications, she really doesn't have any contraindications that I can see. " (RPh04, diabetes) 2) "I'm asking, you know, any history of thyroid cancer in your family, any sort of history of pancreatitis, gallstones. Stuff like that. I'm gonna ask that for that, like it's rare, but still as part of the prescribing process" (RPh05, diabetes) 3) "definitely not a beta blocker because he has asthma." (RPh03, hypertension) |
| Treatment planning | Cost and coverage | Treatment is on provincial (public payer) formulary. Co-pay treatment is acceptable | 1) "It's generally covered by all third party plans, so it was, but you know honestly was that first top of mind? No. because I think you know pretty much they're all they're all relatively affordable and they're all relatively covered by third party plans." (RPh07, hypertension) 2) "where most people are financially, better off than most, and so there are more likely to pay for it out of pocket, even if their insurance denies it." (RPh08, diabetes) 3) "because if they can't afford it, they're obviously not gonna take it." (RPh04, diabetes) 4) "So if she is covered by seniors primary care in terms of SGLT2 inhibitors that are covered, we have dapagliflozin which is full benefit, but then also Jardiance or empagliflozin, which is exception status but she would meet the exception status for it." (RPh03, diabetes) |
| Evidence and experience | Evidence or lack of evidence for efficacy | The clinician's understanding of the evidence (or lack of evidence) of therapeutic effect for defined outcomes | 2) "So I know that there are studies conducted specifically in otitis media that have demonstrated superiority of ibuprofen over acetaminophen, or NSAIDs over acetaminophen." (RPh01, pediatric fever) 2) "I mean and just the results are like fairly spectacular with those drugs and, it doesn't, there's not a lot of drugs that really kind of like impress me usually lately." (RPh04, diabetes) 3) "We're not gonna do a head-to-head trial of ARB versus ARB because we would need a sample size bigger than this planet." (RPh01, hypertension) 4) "I perceive the SGLT 2s having more of a renal benefit. But I know that we are seeing some renal benefit with the GLP 1s, but not as much for saying more like MACE, cardiovascular benefit, from what I understand of the evidence." (RPh05, diabetes) |
| Evidence and experience | Evidence of side effects and tolerability | The clinician's understanding of evidence (or lack of evidence) of treatment harms | 1) "in my head, they seem to be equally like safe or equally like the the risk for hypoglycemia is the same." (RPh08, diabetes) 2) "talk about side effects, I mean both fairly well tolerated in the short term.' (RPh04, pediatric fever) |
| Evidence and experience | Experience as evidence | Clinician draws on their own experiences using a medication as a source of efficacy or safety evidence. | 1) "I've seen A1C's go from like 10 to like 7… So from my experience like this works" (RPh08, diabetes) 2) "I guess I have more experience. Honestly, personally with perindopril it certainly does the trick, so that probably influenced" (RPh07, hypertension) |
| Evidence and experience | Evidence strength or robustness | The clinician's judgement or critical appraisal of the strength/weakness of evidence and robustness/limitation of evidence regarding an efficacy or safety outcome | 1) "From what I understand is we have some evidence for some of the GLP 1s, but not all of them yet. Like there are assuming it's gonna be a class effect that I still think we're waiting on a little bit more information." (RPh05, diabetes) 2) "It also is great at reducing blood sugars, but we don't know. It's so new... we don't know the long term side effects" (RPh06 diabetes) 3) "Then I haven't really read any specifically current recommendations or even information or comparisons, so that would be one I would probably need to go look at a little bit more, but you know they pretty much seem to be pretty much on par." (RPh07 hypertension) |
| Evidence and experience | Evidence generalizability and applicability | The clinician's judgement of the ability or inability of being able to apply evidence to the patient case or extrapolating evidence beyond populations | 1) "my previous experience and expectation of pain control of acetaminophen versus ibuprofen in other populations are likely to extrapolate to this population.” (RPh08, pediatric fever) |
| Drug-related factors | Route of therapy | General acceptability of medication route of administration influences choice (oral, SC, IM, rectal) | 1) "but you know, let's try to avoid the price and the needle." (RPh07, diabetes) 2) "So she has no preferences for administration. I'm assuming that means like pills versus needles versus all that stuff." (RPh05, hypertension) 3) "generally it would be oral in this particular instance, although I guess the one that if there was, you know vomiting acetaminophen is available in a rectal formulations, then I might consider that at the at the child wasn't able to, wasn’t keeping the meds down." (RPh04, pediatric fever) |
| Drug-related factors | Formulation | Assumed or acknowledged acceptability of medication formulation influences choice (tablet, liquid, suspension, extended release, etc) | 1) "So they're comfortable with an oral syringe, but I would... ehh a 20 month old. Yeah, probably not gonna do chew tablets." (RPh05, pediatric fever) 2) "would you prefer bubble gum or strawberry like like I think patient preference on flavor of what they'll actually take is pretty key here because there's still little and I know that certain flavors are not liked and liked." (RPh05, pediatric fever) |
| Drug-related factors | Availability / shortage | Availability of a treatment option and history of availability (drug shortages) | 1) "there was a lot of availability issues. Although I will say most people still manage to, at least sporadically have access, and otherwise we switched a number of people to dulaglutide, or some people to oral semaglutide, who were able to pay for it." (RPh04, diabetes) 2) "you know, rural you might be looking at availability a little bit more." (RPh05, pediatric fever) 3) "Yeah, like I'm now traumatized from losartan. Like I I just don't even go there." (RPH08, hypertension) |
| Drug-related factors | Organ function dose or frequency adjustments | Adjusting dose or frequency based on organ function (e.g. renal or liver function) | 1) "I guess the benefit SGLT2s versus GLP1 sort of 1 requiring less sort of tweaking in terms of dose adjustments or sort of cautions of like all I got to make sure that I monitor closely so I stop that medication." (RPh08, diabetes) 2) "So I'm thinking about her GFR is a little bit reduced. Metformin dose, I'm wondering if we are gonna need to modify." (RPh04, hypertension) 3) "And same with the GLP 1 I don't think there's a lot of evidence of stopping them at any point, so those ones we wouldn't need to adjust like until we get to the bitter bitter end." (RPh05, diabetes) |
| Drug-related factors | Drug-interactions | Perceived risk of drug interactions, in general or actual presence of drug interactions based on patient's current medications | 1) GLP 1s, I don't really worry about drug interactions cause there really aren't many of them. (RPh04, diabetes) 2) "It doesn't interact with other meds." (RPh08, hypertension) 3) "I was looking for any drug interactions that I would be concerned about with ibuprofen." (RPh03, pediatric fever) |
| Drug-related factors | Regimen complexity and flexibility | Factors affecting complexity and flexibility of a regimen such as titrating to target doses, tapering, flexibility to use combination products, risks of administration errors due to interval between doses (e.g. acetaminophen vs ibuprofen q4-6h vs q6-8h) | 1) "I think like just cause like with the SGLT 2 it's like a one and done dosing you don't really have to check in with them again." (RPh08, diabetes) 2) "I don't like to do a combo product until they get the the dose sorted. And then if they're having a problem with the hydroclorothiazide if it's in the combination, it's hard to pivot if it's the combination." (RPh06, hypertension) 3) "So if the patient required further blood pressure lowering down the line that could help with adherence or it would just be more convenient for him if he wanted to take a combination pill." (RPh03, hypertension) |
| Drug-related factors | Mechanism of action / pharmacodynamics | Medications preferred that more specifically targets pathophysiology of problem (medical problem or side effect problem) | 1) "Ibuprofen has the anti inflammatory whereas Tylenol is just purely a pain reliever and fever reducer, so ibuprofen would win out as the anti inflammatory option." (RPh07, pediatric fever) 2) "the mechanism of action of otitis media is inflammation and I'm choosing an anti-inflammatory." (RPh03, pediatric fever) |
| Drug-related factors | Duration of action | Clinician considers a preference for drugs with a longer duration of action | 1) "I'm sure insulin's gonna work faster to decrease the blood sugar, but in terms of getting it down overall I think it would be comparable." (RPh06, diabetes) 2) "And it lasts like just couple hours longer, and is magic in the middle of the night." (RPh04, pediatric fever) |
| Drug-related factors | Onset of action | Clinician considers the onset of action in relation to the intended treatment effect. Drugs with a faster onset of action may not be relevant for chronic conditions. | 1) "I mean the SGLT2 is going to be faster onset… but that doesn't really factor in" (RPh04, diabetes) 2) "for sure you're not gonna see your blood pressure drop in in a day or two or anything." (RPh07, hypertension) |
| Drug-related factors | Environmental impact of treatment | Impact of environmental impact on treatment decisions (e.g. packaging, MDI vs DPI) | 1) "Such a waste of packaging, though for dulaglutide. Like, from an environmental point of view, we do enough damage in healthcare. Like I feel like that's really obnoxious. There's like a lot of pens." (RPh04, diabetes) |
| Drug-related factors | Dose efficacy and equivalency | Evaluating whether different doses of the same or different medications within or across drug classes achieve equivalent therapeutic effects, particularly when switching between agents. | 1) "There is one of them that were short and we were trying to figure out how to interchange between one ACE and ARB... I wanna know that you know 4 milligrams of perindopril is gonna be equivalent to whatever might have been kind to start." (RPh07, hypertension) |
| Patient-centered factors | Disease severity | More severe disease presentation necessitates specific treatment interventions (e.g. insulin which carries higher risk) or combination treatment | 1) "I did not consider combination therapy because they were pretty close to the target that I perceived they should be at." (RPh05, hypertension) 2) "So I would have potentially considered a combination therapy if the patient, if the patients blood pressure was quite a bit above target." (RPh03, hypertension) 3) "I usually don't start 2 at the same time right away, unless they’re A1c is like 10 like over 10, or like 12%." (RPh08, diabetes) |
| Patient-centered factors | Disease trajectory and long term chronic and complication considerations | Considering possible complications of uncontrolled disease and risk factors for comorbidities over time based on initial diagnosis (e.g. hypertension diagnosis and risk of chronic kidney disease; diabetes and risk of cardiovascular disease) | 1) "I was thinking more like long term like this guy just got diagnosed with hypertension. There's probably gonna be more issues down the road, so let's get them on something [renal] protective now." (RPh05, hypertension) 2) "I think the blood glucose lowering effects of the flozins decrease as the kidney function continues to decline, but they still have the cardiovascular and renal protective benefit of it." (RPh07, diabetes) 3) "It can help prevent future decline of her kidney function" (RPh03, diabetes) |
| Patient-centered factors | Risks of polypharmacy and prescribing cascades | Recognizing the presence of or avoiding polypharmacy or prescribing cascades | 1) "There's no need to start two drugs.We need to start one and then if we need more, we add more. We don't start with two" (RPh06, diabetes) |
| Patient-centered factors | Patient specific tolerability / side effect preferences | Patient specific comments regarding assessment of overall tolerability including drug cautions that would require monitoring for that specific disease state being treated (e.g. HF and dapa and fluid status) | 1) "For thiazides, the risk is hypernatremia in addition to other things. So where their sodium was a little bit borderline low. I was more concerned about that risk over the risk of hyperkalemia with ACE and ARBs." (RPh03, hypertension) 2) "So my preference would be that we would use an ACE or an ARB over a thiazide like diuretic because he appears that he would tolerate it well, given his age and renal function" (RPh01, hypertension) 3) "I felt she was more likely to go to the hospital with like hypoglycemia versus like a urinary tract infection" (RPh05, diabetes) |
| Patient-centered factors | Actual response to current or previous treatments | Adjustment of options based on therapeutic or safety response to current or previous treatments (including patient noticeable changes such as weight loss, QoL improvement, etc) | 1) "Failure of one of them is probably is when I would have opted for that." (RPh03, pediatric fever) 2) "with all the options for therapy like we would only go to insulin if nothing else was working" (RPh05, diabetes) 3) "And I always say the combination if one agent alone is not sufficient." (RPh06, pediatric fever) |
| Patient-centered factors | Impact of management on comorbid illnesses | Treatment recommendation improves or worsens comorbid illness or patient assessment. | 1) "So he's already experienced an ADR, he has asthma. I do not want to muddy the picture with asthma, I if he gets a new onset cough, he already has a comorbidity that it could, Is this worsening asthma? Is this ace induced cough?" (RPh01, hypertension) 2) "she has knee OA so I'm wondering about her weight. So ideally would like to try to avoid insulin." (RPh04, diabetes) 3) I'm so the fever might be suppressed, but if there's still, like, lethargic, pale like, you know, if there's other symptoms going on, then that's not gonna be masked. Like Tylenol is not gonna mask like a child who is lethargic." (RPh05, pediatric fever) |
| Patient-centered factors | Monitoring burden (patient) | Laboratory investigations required to monitor for safety of medication | 1) "So like, they're like I view like SGLT like a GLP1 says like less need for like monitoring for that. " (RPh08, diabetes) 2) "Like we would need to like that little risk of the hyperkalemia and then the transient increase in the creatinine. So you might need to do a little bit more monitoring on that versus like a diuretic." (RPh05, hypertension) 3) "the continuous blood glucose monitors as sort of mitigating some of that of like that your Dexcom or your Libre" (RPh06, diabetes) |
| Patient-centered factors | Shared decision making / patient preference | Involving patient in shared decision making when additional input or preferences are needed | 1) "So once a week injection versus once a day oral pill umm, I guess it depends on what she'd like." (RPh08, diabetes) 2) " parents especially, are especially scared of fevers. I get it. Fevers can be scary, so if you're concerned, go with both." (RPh06, pediatric fever) 3) "I think in terms of what I would be looking at, as I really try to keep patient preference in mind and what's what's gonna be feasible" (RPh05, diabetes) |
| Patient-centered factors | Explore the interplay between psychosocial context and management | Describing how social history or assessment of patient's health literacy influences decision making, or availability of resources/support at home (e.g. caregivers) | 1) "Depending on it doesn't say like what her home situation is, or what's going on like does she have a partner at home?" (RPh05, diabetes) 2) "you want to make sure that your mom or parent is going to be able to follow that recommendation" (RPh07, pediatric fever) 3) "So if they're not listening or I'm confident they're going to, uh, make an error, I can only give them one." (RPh06, pediatric fever) |
| Patient-centered factors | Age considerations | Age as a risk factor for safety concerns, for other diagnoses, for long term trajectory and common comorbidities | 1) "I think the ages are really a big factor in being quite stable in terms of like other things going on. I feel like usually for a patient like this, you might have mental health concerns or Alzheimer's or something going on." (RPh05, diabetes) 2) "she's 71 years old. So she's older, adult, but not too old or too frail or functionally dependent. No cognitive impairment there. We probably can be a bit stricter with her A1C target." (RPh08, diabetes) |
| Setting influence | Implications of available system resources (office, hospital, community, and inter- and intraprofessional) on diagnostic or management choices. | Clinicians considers system specific use of resources regarding the treatment or monitoring of patients i.e. cost of monitoring, availability of monitoring, HCP access. | 1) "So reality is a lot of people don't have quick access to a to a physician, no matter where you are." (RPh07, diabetes) 2) "I think if you had done an ACE ARB versus a thiazide diuretic, I think I would have been doing labs anyways in two to three weeks to check electrolytes. So whether I was checking for electrolytes and creatinine versus electrolytes, I guess like cost wise checking electrolytes only is gonna be a little bit cheaper." (RPh05, hypertension) |
| Setting influence | Support from clinical decision support systems | Input from external support systems to provide specific drug information as a passive source of information (e.g. flagging drug interactions, selecting drug dose or strength, identifying dosage forms) | 1) "I do rely on computer software, just I think it's just the sheer volume of information we need to have that safety net" (RPh07, diabetes) 2) "Like in my current practice in the prescribing software that I use when I'm prescribing a medication, I always check everything beforehand, like I check the dosage, strength, I check for interactions in a completely different software program, like I get everything all teed up to go until I'm 100% confident that everything that I'm entering is correct." (RPh03, hypertension) |
| Setting influence | External influences on practice | Acknowledging local influences on practice such as local prescribing patterns, physician/NP preferences, acknowledged marketing, and available work time | 1) "I know lots of doctors who choose perindopril. You can just see, you know volume wise how many times a day you're checking a perindopril." (RPh07, hypertension) 2) "AAA: do you think of Ozempic as top of mind because of that practice around you, people also prescribing it around you? RPh08: Yeah, probably." (RPh08, diabetes) 3) "Maybe it's a marketing thing, but Children's Tylenol is what comes to mind first versus like Children's Advil." (RPh05, pediatric fever) |

## Appendix D: Presence of reasoning factors during think aloud and semi-structured interviews.

| Reasoning Theme | Reasoning Factors | Diabetes TA  (n=8) | Diabetes SSI  (n=5) | Hypertension TA  (n=7) | Hypertension SSI  (n=6) | Pediatric fever TA  (n=8) | Pediatric fever SSI  (n=8) |
| --- | --- | --- | --- | --- | --- | --- | --- |
| Evidence and experience | Evidence or lack of evidence for efficacy | 5 | 5 | 3 | 6 | 3 | 6 |
|  | Evidence for efficacy matched to patient goal or prioritized problem | 7 | 5 | 4 | 6 | 3 | 3 |
|  | Evidence for safety (general) | 0 | 4 | 1 | 6 | 2 | 6 |
|  | Evidence of side effects and tolerability | 2 | 3 | 1 | 4 | 2 | 2 |
|  | Evidence applicability | 0 | 0 | 0 | 0 | 0 | 2 |
|  | Evidence strength or (lack of) robustness of evidence | 0 | 3 | 0 | 2 | 0 | 0 |
|  | Clinician experience as evidence | 0 | 3 | 1 | 3 | 0 | 2 |
| Patient centered factors | Disease severity | 3 | 2 | 1 | 3 | 2 | 6 |
|  | Disease trajectory | 1 | 1 | 0 | 2 | 0 | 0 |
|  | Response to current or previous treatments | 5 | 3 | 1 | 4 | 1 | 5 |
|  | Side effects and tolerability (patient specific) | 4 | 4 | 2 | 5 | 0 | 2 |
|  | Impact of management on comorbid illnesses | 4 | 3 | 3 | 6 | 0 | 3 |
|  | Shared decision making | 5 | 5 | 3 | 2 | 3 | 3 |
|  | Limitations of interventions based on pt physiology/limitations | 1 | 0 | 1 | 1 | 0 | 0 |
|  | Monitoring burden (patient) | 0 | 5 | 0 | 5 | 0 | 0 |
|  | Interplay between psychosocial context and management | 1 | 0 | 0 | 0 | 1 | 2 |
|  | Age considerations | 4 | 1 | 2 | 1 | 0 | 0 |
|  | Polypharmacy and prescribing cascade | 0 | 1 | 0 | 1 | 0 | 0 |
| Drug centered factors | Organ function dose or frequency adjustments | 3 | 5 | 0 | 0 | 0 | 0 |
|  | Formulation | 0 | 0 | 0 | 0 | 1 | 2 |
|  | Route of therapy | 5 | 3 | 0 | 0 | 0 | 5 |
|  | Availability or shortage | 0 | 5 | 0 | 4 | 0 | 4 |
|  | Regimen complexity or flexibility | 3 | 4 | 2 | 5 | 2 | 4 |
|  | Dose efficacy and equivalency | 0 | 0 | 0 | 2 | 0 | 1 |
|  | Drug-interactions | 1 | 4 | 1 | 4 | 2 | 2 |
|  | Mechanism of action / pharmacodynamics | 0 | 0 | 0 | 1 | 2 | 6 |
|  | Duration | 0 | 3 | 0 | 2 | 2 | 6 |
|  | Onset of action | 0 | 2 | 0 | 1 | 1 | 4 |
|  | Environmental impact | 0 | 1 | 0 | 0 | 0 | 0 |
